# Production and Analysis of Health Indicators: The Role of Academia

**DOI:** 10.1371/journal.pmed.1001004

**Published:** 2010-11-30

**Authors:** Christopher J. L. Murray, Alan D. Lopez

**Affiliations:** 1 Institute for Health Metrics and Evaluation, University of Washington, Seattle, Washington, United States of America; 2 School of Population Health, The University of Queensland, Brisbane, Australia

**Summary Points**

- Progress on the timely, valid, reliable, and comparable measurement of health indicators requires tools and instrument innovation; enhanced national capacity to collect, process, archive, and share data; norms and standards for indicator definitions and computation; multiple independent analyses of data; and effective translation of results into policy.
- Academia and the associated process of peer-reviewed publication and open debate should play the central role in the creation of new tools and instruments and in the rigorous analysis of existing data to yield the best possible assessments of key health indicators.
- The United Nations and World Bank have important roles to play in helping countries build capacity for data collection and analysis, setting norms and standards, and helping national decision-makers translate evidence into new policy directions.

## Evolving Roles for the State, Supra-National Bodies, and Science

The collection, computation, and publication of health indicators was for much of the 19^th^ century and the first half of the 20^th^ century the business of states—reflecting the etymology of the word “statistics” (from Statistik, or “of the state”). With the spread of independence in the developing world in the second half of the 20^th^ century, the United Nations (UN) began to play a supra-national role in the production of health indicators to bolster weak capacity in some developing countries. Yet, over the past five or six decades, the expectations for the field have expanded considerably. New constructs have been introduced such as cause-specific mortality, quality of care, and functional health status. Measurements must often deal with the simultaneous problems of synthesizing conflicting sources, missing data, instrument bias, substantial non-sampling error in surveys, and missing denominators, particularly in administrative data. Reflecting these complexities, in the last 30 years, the role of the academy has expanded from tools and methods innovation to the production of comparable measurements for key health indicators in various countries [1] and globally [2],[3]. Academic demographers have been involved in the production of comparable population measurements for nearly 50 years [4]. In the last two decades, epidemiology and other health-related disciplines have also been actively engaged in population-level health measurement [5].

Reflecting the progressive diversification of actors in the production of health indicators, alternative statistics are now available from national authorities, from various UN agencies, and from the peer-reviewed scientific literature [6]–[10]. Alternative measurements have engendered considerable concern from health advocacy groups and practitioners who rely on sound measurement to guide programs, and from donor agencies. With the 2015 target looming, interest in reliably monitoring the progress of countries in attaining (or not) the UN Millennium Development Goals has intensified. In this commentary, we consider the potential roles of different actors and highlight four key issues that are widely debated.

## Key Ingredients for Sound Measurement and Policy Translation

There are five distinct areas that contribute to the ultimate production of timely, comparable, valid, and reliable health indicator measurements and their translation into national policy dialogue.

### Tools and Instrument Innovation

The set of tools available for data collection and analysis is rapidly changing. Advances in computing mean that innovations such as electronic medical records or Bayesian measurement models are now feasible even in low-resource settings. Most of the innovation in this area will continue to come from the private sector (including non-governmental organizations) and academia. The role for national statistical authorities or UN and World Bank institutions will likely be restricted to agenda promotion, advocacy for application, and financing.

### Capacity to Collect, Process, Archive, and Share Data

Whether in a developed or developing country, good health measurement requires capacity in national statistical offices and Ministries of Health to collect administrative data on case reporting, service delivery and expenditures, and household data through surveys and censuses. Capacity to collect, process, and archive is both human and physical. Our view is that academia has an important role in providing appropriate training and education; national governments a critical role in nurturing this capacity at national, regional, and local levels; donor agencies a role in helping low-income countries finance capacity development; and the UN system a role in advocating for capacity development and helping broker financial and technical assistance. The success of the United Nations Children's Fund (UNICEF) in raising funding for the Multiple Indicator Cluster Surveys is a clear example where this role has been extremely beneficial. Harnessing the capability of scientists within countries and around the world for data analysis requires archiving and sharing data in a way that protects confidentiality but stimulates innovation.

### Norms and Standards for Health Indicators

Given the rapid innovation in tools and instruments and the expanding set of constructs for measurement, comparable measurement requires a periodically revised set of nomenclatures, definitions, and data standards. Non-governmental organizations or public–private partnerships have been effective in promulgating some standards such as Health Level Seven (HL7) or Systematized Nomenclature of Medicine—Clinical Terms (SNOMED CT). Nevertheless, there is a key role for supra-national bodies such as the World Health Organization (WHO), given its governance mandate, in setting universal standards. WHO has successfully stewarded the International Classification of Diseases for over 60 years and should continue to be a leader in setting a wide range of health norms and standards.

### Analysis of Data

It is the nature of health data that estimation of health indicators often requires more than data processing. Measurements from different sources may conflict, data for some localities or years may be missing, survey instruments may be biased, administrative data systems may exclude groups such as the poor, and so on. After processing, data need to be analyzed with the best available scientific methods. Analysis is required both for the production of comparable, valid, and reliable measurements as well as for the more difficult task of interpretation or causal inference. National statistical authorities, academics, and UN institutions all undertake data analysis. Given the myriad data challenges outlined above, there is considerable scope for reasonable scientists to disagree on the interpretation of data, whether for descriptive or causal reasons. Given that the task is fundamentally a scientific challenge, we believe that it should primarily be the role of independent scientists in academic institutions, independent of the agencies or programs that collect or collate the data. Additionally, the discipline of peer review, although imperfect, brings a transparency and objectivity to the analysis of data.

### Policy Translation

Sound measurement of health indicators should serve as input to a more informed national health policy debate. The gap between science and policy formulation can be large. Through their trusted relationship with governments, UN and World Bank institutions can play a key role in policy translation. In some countries, independent think tanks, academics, and national academies of science can also play a key role in the translation of empirical findings into policy options.

## Four Issues in the Debate

### 1. Estimates or Country Reporting?

The debate over the best measurements for child and maternal mortality is sometimes framed as a debate on estimates versus country reports [11]. Proponents for the latter appear to believe that “estimates” are inherently undesirable and fraught with uncertainty, while country reports are inherently valid, reflecting the reality on the ground. This debate masks two different issues. First, in 2010, all sound measurements are in some way “estimates” in that appropriate statistical methods have been applied to the primary processed data to yield a measurement. For example, in a high-income country like the United States, measurement of the maternal mortality rate must deal with misclassification of maternal deaths to other causes, census counts need to be adjusted for under-enumeration, and smoking rates from the Behavioral Risk Factor Surveillance System must be adjusted for nonresponse bias by age, sex, and race.

The second issue is one of political provenance. For some health indicators, it is awkward for some UN agencies to disagree with national authorities. This debate is code speak for avoiding disagreeing with Ministries of Health or other government authorities by publishing government figures regardless of the quality of data or the rigor of the analysis applied to the data. The real issue is whether we want science-based measurement or political measurement. The world needs the former; there really is no role for the latter.

### 2. Multiple Figures Will Confuse Donor Country Agencies and Legislators and Decision-Makers in Low-Income Countries

Some advocacy groups believe that public debate over the magnitude and trend of key health indicators can undermine support for health programs among donors and will confuse leaders in developing countries. For example, in the maternal mortality debate, one advocate argued that scientists should be locked in a room until they agree on one set of numbers. We believe the drive to artificial consensus is fundamentally misguided. Health leaders in developing countries have the sophistication and experience to understand that measurement is challenging and that there will be uncertainty in the assessment of levels and trends. The recent unfolding of “Climategate” illustrates how forced consensus can undermine public confidence in an important agenda [12]. Rather than forcing a false consensus among scientists, we believe that it is the responsibility of the scientific community to encourage robust public discourse on critical health indicators; such dialogue will lead to both a better understanding of the health challenges nations face and an appreciation of the realities of uncertainty in measurement. It ought also to accelerate efforts in countries to improve critical health information systems.

### 3. The UN as Player and Referee in the Scientific Arena

Some excellent scientists work for the UN and its technical agencies. In their role as scientists they sometimes analyze data and produce measurements for health indicators. Occasionally, this work is subject to the normal process of peer review, but more often it is published under the imprimatur of the UN. The global public health and development communities need the UN, especially WHO, to assume the leading role in setting norms and standards for indicators. It is difficult to see how the UN can be the trusted developer of norms and standards and a respected neutral broker when it is also trying to compete in the health measurement arena [13]. Inevitably, this creates a conflict of interest, often leading to UN agencies promoting their own estimates using their imprimatur, depriving countries and other users of the advantages of scientific exchange and debate. In our view, these efforts progressively undermine the UN's capacity to set standards in the long term. The UN can, and we believe should, use their effective relationships with governments to promote the translation of scientific findings into more effective national policy formulation. Can one clearly distinguish data analysis to produce timely, valid, and reliable health indicator measurements from analyses to support policy translation in a given country? One useful guide to the dividing line is that when there is a need for innovation in methods or application of methods, the role for academia is more compelling. Timely application of existing analytical tools to inform policy as part of an ongoing institutional dialogue may be more effectively delivered by the UN institutions, national think tanks, or consulting organizations.

### 4. Good Practice Guidelines

The rise of low-cost computational power has already had a far-reaching impact on the analysis of data to produce comparable, valid, and reliable measurements of health indicators. An impressive array of computationally intensive methods has now been applied in health [14], with further innovation and methodological advances on the horizon. Given the methods innovation and the increasingly diverse set of scientists drawn to the challenge of health measurement, it is becoming increasingly important to create a set of guidelines for health indicator studies. These guidelines should cover key areas such as the definition of a systematic analysis using all available data, requirements for transparency on data and methods, necessary tests of predictive validity, calculation of uncertainty intervals based on sound scientific principles, and attempts to adjust data for known bias.

We believe that the diversification of actors involved in health indicator measurement over the last 30 years or so has been tremendously positive. The expanding role of academics in health measurement, whether at the national or global level, is helping to shift practice away from a political state-based model of health measurement to a science-based model. As analytical capacity in academic institutions in developing countries continues to expand, more national and regional comparative studies will emerge from scientists at these institutions. This is a most welcome development, and efforts to strengthen analytical capacity must remain a priority [15]. There are important roles for states, inter-governmental organizations, donor agencies, the private sector, and academia. By harnessing the extraordinary power of innovation in the scientific world, decision-makers in all countries will benefit from more timely, comparable, valid, and reliable measurements of the health constructs that they seek to influence.

## Abbreviations

WHO: World Health Organization
